# Case report: Giant cell tumor of bone in the mandible of a goat—diagnostics, surgical treatment, and outcome

**DOI:** 10.3389/fvets.2024.1457223

**Published:** 2024-08-08

**Authors:** Nora M. Biermann, Susanna Piechl, Nora Dinhopl, Andrea Fuchs-Baumgartinger, Christiane Weissenbacher-Lang, Christof A. Bertram

**Affiliations:** ^1^Clinical Department for Small Animals and Horses, University of Veterinary Medicine Vienna, Vienna, Austria; ^2^Department for Farm Animals and Food System Science, University of Veterinary Medicine Vienna, Vienna, Austria; ^3^Department of Biological Sciences and Pathobiology, University of Veterinary Medicine Vienna, Vienna, Austria

**Keywords:** mandibulectomy, neoplasia, large animal, surgery, oral cancer, giant cell tumor (GCT), head surgery

## Abstract

Neoplastic processes of the mandible and their treatment are rarely reported in large animal species. Specifically, giant cell tumor of bone is an uncommon tumor in animals and has been associated in humans with locally invasive behavior and a high recurrence rate. En-bloc resection is the treatment of choice, but depending on the localization of the tumor, this may result in functional deficits. This report details the diagnostic work-up, treatment, and long-term outcome of a giant cell tumor of bone involving the rostral mandible and mandibular symphysis of a goat. Extensive rostral mandibulectomy involving the entire mandibular symphysis without surgical fixation of the hemimandibles was performed. Histological and electron microscopic findings of the tumor were consistent with a giant cell tumor of bone. Although a mutation of the H3F3A gene is considered the driver of tumor development in human giant cell tumors, using molecular analysis, this gene mutation could not be confirmed in this case. Follow-up examinations revealed spontaneous secondary fusion of both hemimandibles and no signs of tumor recurrence. Nearly 1 year after surgery, the owners reported no signs of tumor regrowth.

## 1 Introduction

The main indication for surgical intervention at the level of the rostral mandible in large animals include trauma, developmental disorders, and neoplastic processes (1). Trauma seems to be most common, but neoplasia has also resulted in treatment by mandibulectomy both in horses and large ruminants (1–4). In goats, only a few reports exist, detailing various neoplastic processes of the mandibles such as lymphosarcoma, fibrosarcoma, non-ossifying fibroma, and osteoma, with treatment either not attempted or unsuccessful (5–9). One case of a goat with a giant cell tumor of bone has been previously described (10), but no treatment was attempted, and the animal was euthanized due to a presumed poor prognosis.

Giant cell tumors of bone develop within the bone and are characterized histologically by osteoclast-like multinucleated giant cells and fibroblast-like spindle cells, the latter considered the neoplastic cell population likely arising from the osteoblastic lineage (11). Histologic examination of this tumor requires differentiation from giant cell-rich osteosarcoma and gingival peripheral giant cell granuloma. Giant cell tumors of bone account for up to 20% of bone tumors in humans but are rarely reported in animals (12, 13). In domestic animals, these tumors are most commonly reported in cats (14–16), less commonly in dogs (13), and infrequently in horses (17) and goats (10). They display mostly locally aggressive behavior with a high recurrence rate in humans (18). Rarely, cases of extraskeletal metastasis have been described in both humans and animals (15, 19). In human giant cell tumors of bone, a mutation of the H3F3A gene is considered the driver of tumor development (20); in animals, this mutation has, to the author’s knowledge, not been evaluated to date.

Surgical treatment of mandibular tumors generally involves en-bloc resection of neoplastic tissue with or without internal fixation and is considered the treatment of choice (3, 21, 22). However, descriptions of responses to treatment and long-term outcomes in food- and fiber-producing animals are scarce (3, 4).

This case report aims to describe diagnostics, surgical treatment, and follow-up of a giant cell tumor of bone in the mandible of a young goat.

## 2 Case description

### 2.1 Case presentation

A 2-year-old female pygmy goat (23 kg) presented with a mass on the rostral mandible, initially suspected to be an abscess. Despite conservative treatment and attempted drainage by the referring veterinarian, the mass slowly increased in size in the 2 months prior to referral without affecting food apprehension or chewing.

**Figure 1:**
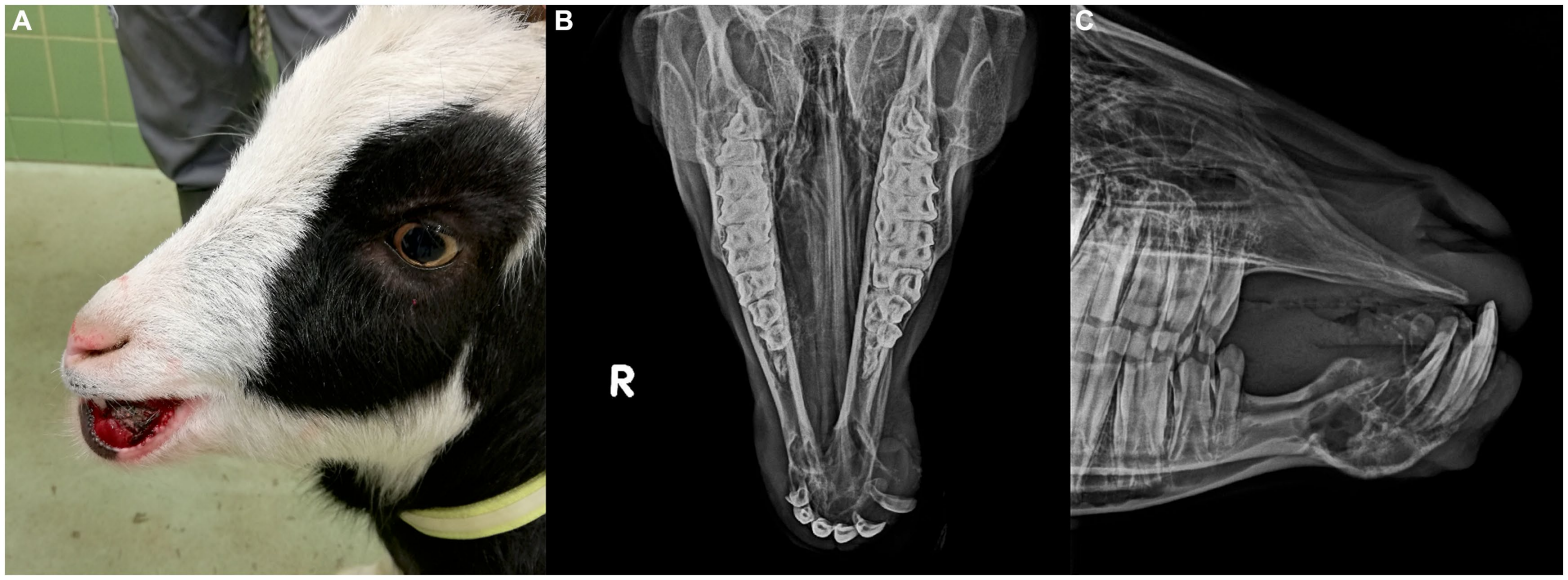
**(A)** Preoperative image of 2 year-old pygmy goat with a giant cell tumor of bone involving the rostral mandible. **(B)** Frontomandibular and **(C)** lateral radiographs of the rostral mandible of a goat with a giant cell tumor of bone. Displayed is an expansile, multi-cavitated mass mostly within the mandibular cortex, with primarily signs of lysis involving the mandibular symphysis. The left mandibular incisor teeth are displaced and partially covered by protruding soft tissue.

Upon presentation, physical examination parameters were within normal limits, and the goat was in good body condition. An approximately 30 mm × 25 mm × 30 mm firm, non-painful mass displaced the left mandibular incisor teeth, with the left mandibular third incisor tooth being highly mobile. The mass was mostly covered by partially ulcerated oral mucosa and firmly adhered to the mandible (Figure 1A). A fine needle aspirate, taken after local anesthesia, was non-diagnostic.

Radiographs showed an expansile, multi-cavitated mass within the rostral mandibular cortex, including the symphysis, extending caudally approximately halfway toward both mandibular second premolar teeth. Marked signs of alveolar bone resorption with lateral displacement of the left mandibular incisor teeth which were partially covered by protruding soft tissue, was present (Figures 1B,C).

Mandibular and cervical lymph nodes were not enlarged, and thoracic radiographs showed no evidence of metastatic lesions at the time. Hematologic and serum biochemical profiles were considered normal except for a mild hypoproteinemia (5.6 g/dL; reference interval: 6.0–7.8 g/dL). En-bloc resection of the tumor, including the mandibular symphysis, was recommended. Stabilization of the mandible with an implant (21) was discussed with the owners. However, due to the high risk of infection, the need for a second surgery to remove the implant and financial constraints, a decision was made against fixation.

### 2.2 Surgical treatment and postoperative care

Preoperatively, the goat received Carprofen (1.5 mg/kg, SC) and Amoxicillin (15 mg/kg, IM). After sedation with Xylazine (0.1 mg/kg, IV) and Butorphanol (0.2 mg/kg, IV), general anesthesia was induced with Ketamine (5 mg/kg, IV) and maintained with Isoflurane in oxygen and positive pressure ventilation via an orotracheal tube. The goat was positioned in sternal recumbency, and the rostral mandible was prepared for aseptic surgery. Intraoral bilateral mandibular nerve blocks with 1.5 mL of Procaine (2%) each were performed as described in small animals and horses (23, 24). A circumferential incision was made around the rostral mandible, 0.5 cm caudal to the mass. The incised tissue was sharply separated from the mandibular bone and retracted caudally to expose both mandibular rami. Bleeding during subcutaneous dissection was controlled with electrocautery and manual pressure. A rostral mandibular osteotomy was performed using an oscillating saw, 2 cm rostral to each mandibular second premolar tooth. Again, bleeding was encountered from both mandibular stumps, which could only be controlled with repeated use of electrocautery and manual pressure. The goat lost approximately 15% (approximately 300 mL) of its estimated total blood volume until bleeding was controlled. Repeated measurements of packed cell volume and total solids (PCV/TP) perioperatively continued to be within normal limits, and the goat remained cardiovascular stable under general anesthesia, with additional intravenous fluid therapy.

The subcutaneous tissue and oral mucosa were apposed to cover the mandibular stumps using simple interrupted sutures and 2-0 Monosyn (Glyconate absorbable monofilament, Braun Surgical, Rubin, Spain) in two layers. During recovery from general anesthesia, the goat started to repeatedly vocalize, and bleeding reoccurred. This resulted initially in increasing subcutaneous swelling and subsequent dripping of blood. The goat was re-sedated (Xylazine; 0.07 mg/kg, IV) and manual pressure at the area resulted in hemostasis, after which recovery was successfully continued without the need for a blood transfusion.

The rostral mandible, including the tumorous tissue, was submitted for histopathology in 4% formalin.

**Figure 2:**
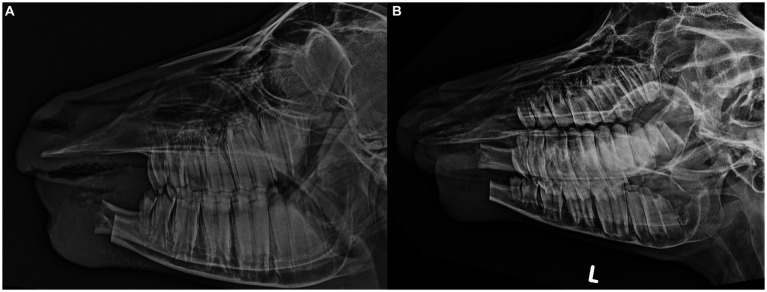
Lateral **(A)** and oblique **(B)** postoperative radiographs after extensive rostral mandibulectomy without internal fixation of the mandibles in a goat to achieve en-bloc resection of a giant cell tumor of bone. A slightly irregular bone structure is visible at the level of the right mandibular stump likely caused by the repeated attempts to achieve hemostasis during surgery.

Postoperatively, the goat continued to receive anti-inflammatory medication (Carprofen 1.5 mg/kg, SC) for 8 days and antimicrobials (Amoxicillin, 15 mg/kg, IM) for 3 days. Additional pain control was provided with a constant rate infusion of Ketamine (3 mcg/kg/min) and boluses of Butorphanol (0.1 mg/kg, IV q 4 h) for the initial 48 h, at which point the goat started to eat both pelleted feed as well as short-stemmed hay. Butorphanol (0.1 mg/kg, SC q 6 h) was continued for an additional 48 h, followed by a gradual decrease and discontinued 7 days after surgery. At this point, the goat showed normal food intake despite the independent movement of both mandibles. No active bleeding occurred postoperatively and swelling of the surgical site remained stable. Radiographs 10 days post-surgery were unremarkable except for a slightly irregular bone structure of the right mandibular stump, likely caused by the repeated attempts to achieve hemostasis during surgery (Figures 2A,B). The surgical site was healing well, with sutures in place and no signs of infection. The goat was discharged with instructions to monitor normal food and water intake, check the surgical site for increased swelling or discharge, and attempt to prevent jumping and headbutting with other goats wherever possible.

### 2.3 Gross and histologic examination

**Figure 3:**
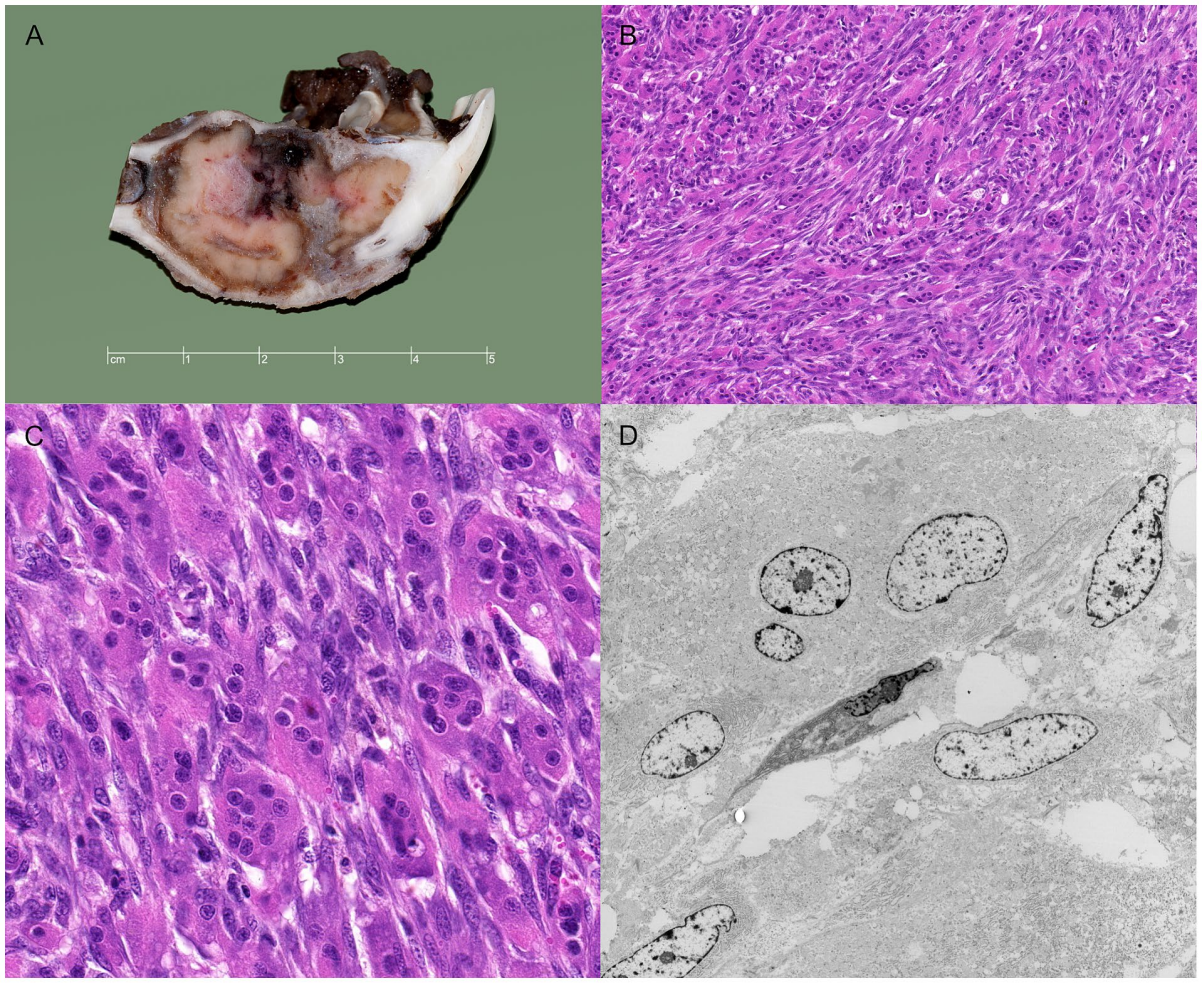
Gross, histologic and electron microscopic images of a giant cell tumor of bone in a goat. **(A)** Gross image showing the neoplastic mass within the bone marrow of the mandible associated with osteolysis and expansion of the bone cavity. The tumor breaks through the bone lingually and protrudes into the oral cavity. **(B,C)** Histological images of the tumor showing the characteristic osteoclast-like giant cells and fibroblast-like spindle cells. **(D)** Electron microscopic image of a multinucleated giant cell (upper part of the image) and spindle cells (lower part of the image).

The bone specimen received from surgery was sectioned longitudinally with a band saw. Grossly, the bone cavity was focally extended by an approximately 4 cm in diameter mass (Figure 3). The cortex of the mandibular bone was attenuated and focally perforated by the infiltrative mass, which formed a 15 mm large extension lingually into the oral cavity. The masses were firm, tan and lobulated.

Thin pieces of tissue (approximately 2 mm thick) were sectioned with the band saw and, after additional fixation in 4% formalin for 24 h, processed routinely to create hematoxylin & eosin-stained histologic sections. Sections that contained cortical bone were decalcified for 24 h in a commercial fluid (Decal®, Quartett GmbH, Berlin, Germany) containing deionized water (90%), hydrochloric acid (<10%), and ethylenediaminetetraacetic acid (<0.1%). Histologically, the highly cellular tumor was characterized by bundles of fibroblast-like spindle cells regularly interspersed with numerous multinucleated giant cells that usually contain between 5 and 15 nuclei. There was minimal fibrous stroma. The oral tumor extension contained multifocal, chronic hemorrhages with multiple hemosiderophages. There was no evidence of osteoid production except for the spicules of reactive bone arising from the cortex. The fibroblast-like spindle cells had solitary elongated, blunt-ending nuclei and moderate eosinophilic cytoplasm with indistinct cell borders. This cell type had low malignancy criteria, including a low nuclear pleomorphism, low mitotic activity (maximum mitotic count in 2.37 mm^2^ was 3), and no atypical mitotic figures. The giant cells had uniform, round nuclei randomly distributed within the abundant, eosinophilic cytoplasm. The histological tumor-free distance between the tumor and the caudal surgical margins was 3 mm.

The histological findings of the present tumor were consistent with a giant cell tumor of bone. The first differential diagnosis was a giant cell-rich osteosarcoma, which, however, seemed unlikely as there was no evidence of osteoid production from the tumor cells and there were minimal malignancy criteria, i.e., no mitotic figures, particularly no atypical mitotic figures, and minimal nuclear pleomorphism of the mononuclear cells (11). The second differential diagnosis was a peripheral giant cell granuloma, which arises from the gingiva and usually does not involve the underlying bone (25). In the present case, the main tumor mass was within the bone marrow indicating that it had arisen from the bone and not gingiva.

### 2.4 Electron microscopic examination

For electron microscopic examination, tissue samples of the tumor were fixed in 5% glutaraldehyde (Merck, Darmstadt, Germany) diluted in 0.1 M phosphate buffer (Sigma-Aldrich, Vienna, Austria; pH 7.2) at 4°C for 4 h. Afterward, samples were post-fixed in 1% osmium tetroxide (Merck) diluted in the same buffer at 4°C for 2 h. After dehydration in an alcohol gradient series and propylene oxide (Merck), the tissue samples were embedded in glycid ether 100 (Serva, Heidelberg, Germany). The ultrathin sections were cut on a Leica Ultramicrotome (Leica Ultracut S, Vienna, Austria), stained with uranyl acetate (Sigma-Aldrich) and lead citrate (Merck). Ultrathin sections were examined with a Zeiss TEM 900 electron microscope (Carl Zeiss, Oberkochen, Germany) operated at 60 kV.

Consistent with the histologic findings, the ultrastructural investigation of the tumor showed predominantly reactive osteoclast-like multinucleated giant cells as well as proliferative mononuclear stromal cells (Figure 3). The stromal cells showed nuclei with an irregular membrane, one prominent nucleolus, as well as condensed, heterochromatin marginal clusters in contact with the nuclear envelope. The cytoplasm of the stromal cells had prominent profiles of rough endoplasmatic reticulum in which flocculent material was present.

### 2.5 Molecular examination

We investigated whether a mutation of the H3F3A gene, the driver mutation in humans, is present in this goat. Ten FFPE tissue sections of 10 μm slice thickness each were deparaffinized according to the modified protocol of Weissenbacher-Lang et al. (26). The modification consisted of using (R) (+) Limonene (SAV Liquid Production, Flintsbach am Inn, Germany) instead of xylene as a solvent. The pellets were stored at 6°C for a maximum of 1 day until nucleic acid extraction. DNA was extracted using the QIAamp DNA Micro kit (Qiagen, Vienna, Austria) according to the manufacturer’s instructions. The PCR mixture for the amplification of the H3F3A gene consisted of 12.5 μL Kapa 2G Fast HotStart ReadyMix (Merck, Vienna, Austria), 0.4 mM of each primer (fw: 5′-TGGCTCGTACAAAGCAGACT-3′, rv: 5′-ACGGAGTGCCACAGTACCAG-3′), 1 μL MgCl_2_ (Peqlab, VWR, Vienna, Austria), 1 μL template DNA (1:100 dilution), and distilled water to a total volume of 25 μL per reaction. The cycler program started with a heat denaturation step at 95°C for 3 min, followed by 40 cycles at 95°C for 15 s, 60°C for 15 s, and 72°C for 25 s, and terminated with an extension step at 72°C for 1 min. Gel electrophoresis was conducted as described previously (20). The positive PCR product with an amplicon length of 149 bp was purified using the MinElute PCR Purification kit (Qiagen, Vienna, Austria) and submitted for Sanger DNA sequencing (Microsynth, Vienna, Austria). Sequences were assembled with the software BioEdit Sequence Alignment Editor 7.1.3.0 (27). The sequences were submitted to the NCBI Basic Local Alignment Search Tool (BLAST) and aligned with the published mRNA sequence of a *Capra hircus* H3.3-like histone (accession number XM_018048046) to investigate a suspected G > T transversion of the H3F3A gene.

**Figure 4:**
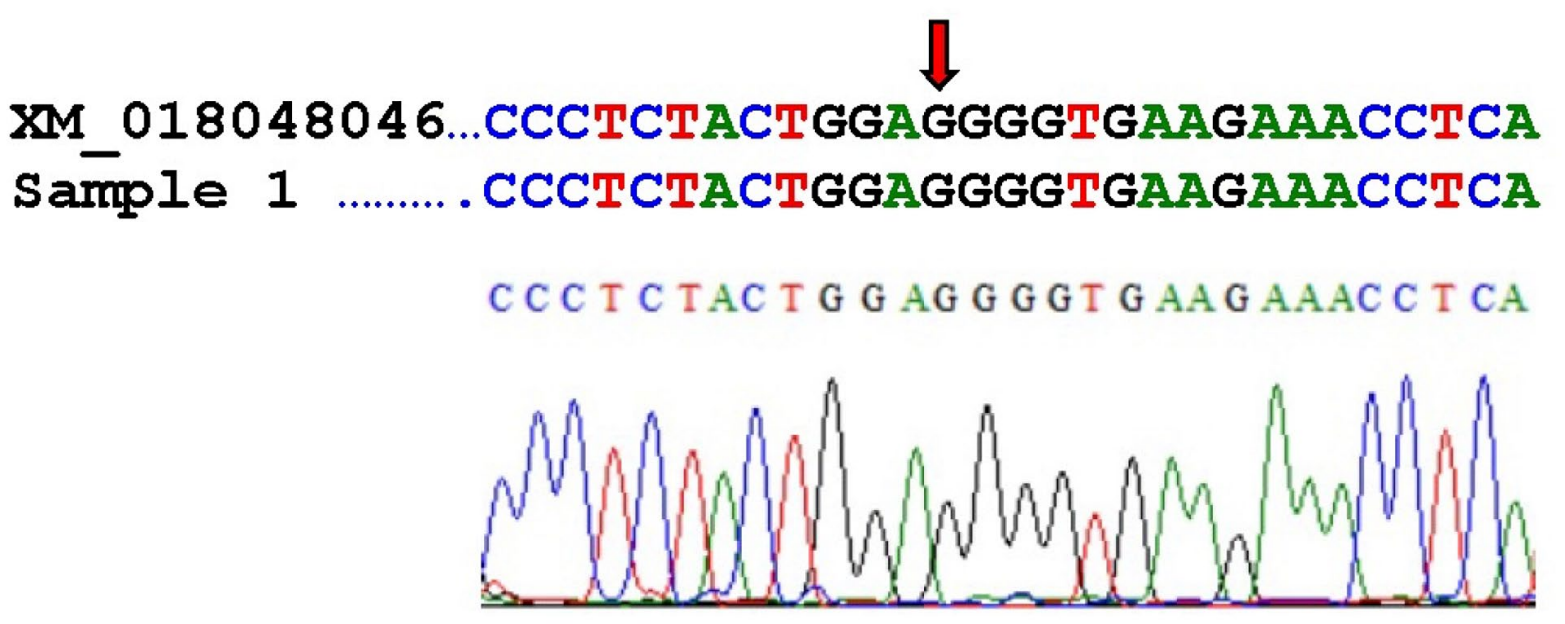
Alignment of the published mRNA sequence of a *Capra hircus* H3.3-like histone (accession number XM_018048046) with our sequence (referred to as Sample 1) and electropherogram of our sequence. The arrow indicates the transversion site.

The G > T transversion of the H3F3A gene could not be confirmed in the present case. Figure 4 shows the alignment of the published mRNA sequence of a *Capra hircus* H3.3-like histone (accession number XM_018048046) with our sequence. The transversion site of the mutation in human giant cell tumors of bone is marked with an arrow, and our sequence corresponds to the wild type.

### 2.6 Follow-up and outcome

Six days post-discharge, the goat was re-admitted with acute neurological signs, including dull mentation and opisthotonus. Trauma was suspected as the goat was housed with another pygmy goat. Physical examination and blood work were normal except for a mild intermittent opisthotonus that the goat displayed in the first hours of hospitalization, the surgical site was healing well. The goat was treated for presumed head trauma with Carprofen (1.5 mg/kg, SC) and observed in the hospital for the next couple of days, displaying no further neurological signs.

**Figure 5:**
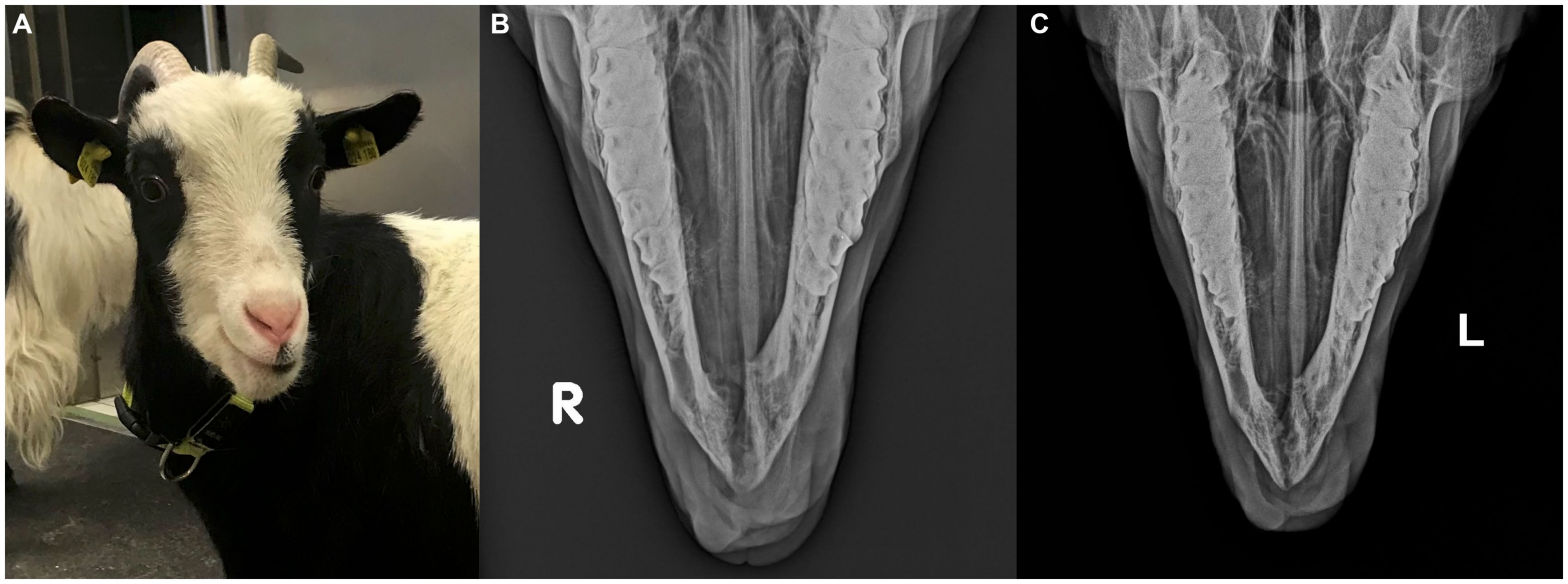
**(A)** Image of the goat 3 months after surgery. At this time, the goat was eating normally, with stability of both mandibles and a firm, prominent bony callus at the surgical site. Cosmetic appearance was deemed satisfactory by the owners. **(B,C)** Frontomandibular radiographic views 3 and 4 months after extensive rostral mandibulectomy without internal fixation of the mandibles. Advanced fusion of both mandibles with moderately irregular bony callus is visible after 3 months **(B)** which appears slightly less irregular with decreased callus size after 4 months **(C)**.

At the 3-month re-check, the goat was eating normally, with stability of both mandibles and a firm, prominent bony callus at the surgical site (Figure 5A). Radiographs showed advanced fusion of both mandibles with moderately irregular bone structure (Figure 5B).

A second re-check examination 1 month later revealed that the callus size had decreased, and bone structure appeared more regular (Figure 5C). The goat was discharged with the recommendation to return for a re-check examination in 6 months and frequent monitoring of the area by the owners for any signs of tumor recurrence in the meantime.

Upon telephone follow-up 10 months after surgery, the goat was reported to be doing well with no signs of tumor re-growth, and the owners were satisfied with the outcome.

## 3 Discussion

This case report describes the successful treatment and long-term outcome of a giant cell tumor of the rostral mandible in a goat. In current literature, there is one report of a giant cell tumor also affecting the mandible; however, no treatment was pursued (10). In the present case, en-bloc resection without fixation resulted in spontaneous fusion of the mandibles 3 months post-surgery, with no tumor re-growth. Neither treatment by extensive rostral mandibulectomy nor short- and long-term response of en-bloc resection of this type of tumor have been previously reported in food-producing animals.

Giant cell tumors of bone frequently originate in the epiphysis of long bones in humans, particularly the distal femur, proximal tibia, distal radius, and sacrum (12, 28, 29), but can also affect the mandible (29–31). These tumors account for up to 20% of bone tumors in humans (12) but are rare in veterinary species (13–16). In large animals, reports are scarce, and information on treatment and outcomes is limited (10, 17).

In this case, the tumor involved the mandibular symphysis, which was possibly the point of origin in this young animal and, therefore, bears similarities to cases previously described in humans (12, 28). However, the driver mutation of the H3F3A gene characteristics for human giant cell tumors of bone was not detected in this goat. Further studies are needed to evaluate whether animal giant cell tumors of bone share a similar oncogenesis as they do in humans. A more in-depth molecular and immunohistochemical comparison of human and animal giant cell tumors is warranted since veterinary literature has extrapolated information from humans due to the paucity of reports in animals (11).

These tumors, though locally aggressive, rarely metastasize (15, 18, 19). Due to their locally aggressive behavior and high recurrence rate with conservative management, en-bloc resection is the preferred treatment in humans (32). The involvement of the symphysis necessitated extensive rostral mandibulectomy to achieve tumor-free margins and prevent further spread in this case.

Extensive rostral mandibulectomy has been reported in small animals, cows, and horses (20, 21, 33, 34). Although goats have been used to study mandibular reconstruction and osteogenesis (35, 36), this specific surgical technique, its complications, and outcomes in goats have not been documented.

Since removal of the entire symphysis results in instability of the mandibles and independent jaw movement as fixation was declined. The owners were informed that this surgical technique may result with time in degenerative changes of the temporomandibular joints (TMJs) as reported in dogs (37). Additionally, since this technique had not been previously reported in goats, other short- and long-term complications could not be entirely excluded.

The primary intraoperative complication was moderate to severe hemorrhage from the surgical site. The well-vascularized tumor received blood mainly through the intra-cortical vasculature within the mandible’s medullary cavity. This became immediately apparent upon the first incision and intensified with the dissection of the mandibles.

Due to the goat being classified as a food-producing animal, certain hemostatic agents, like collagen sponges and antifibrinolytic drugs (e.g., tranexamic acid), could not be used, limiting hemostasis to electrocautery and manual pressure. This resulted in significant blood loss by the end of recovery (approximately 20–25% of the patient’s blood volume). A blood transfusion was considered intra-operatively, but since at that point bleeding was controlled no blood was collected from the goat’s companion. Likely, it would have been helpful during recovery, which was prolonged, because every time the goat started to wake-up, it was vocalizing continuously, which increased cranial blood pressure and resulted in the recurrence of hemorrhage. Re-admission of the goat 6 days post-discharge due to signs of head trauma underscores the point that head surgery in goats comes with species-specific challenges. Ultimately, this goat recovered well from the procedure although it has to be noted that multimodal pain management in the early postoperative period was crucial to promote adequate food intake.

Temporomandibular joint disease, reported in humans and small animals with chronic malocclusion or mandibular instability (36, 37), is a potential long-term complication for this goat. At the last re-check, 4 months post-surgery, radiographic evidence of TMJ disease was not assessed. In a study on dogs with experimentally induced TMJ instability, radiographs did not show osteoarthritis at 3 and 6 months, although histopathology confirmed it (37). TMJ osteoarthritis is rarely reported in food-producing animals, and the long-term impact of altered mandibular alignment on TMJ function remains unknown.

Another possible long-term complication might be tumor recurrence with an unknown recurrence rate due to the limited number of reports in veterinary species. Re-check examination and radiographs did not indicate tumor recurrence at the time, and at telephone follow-up nearly 1 year after surgery, the goat was healthy with no signs of regrowth. Despite a recommendation for a follow-up appointment with control radiographs, the owners declined due to the goat’s good overall health.

Extensive rostral mandibulectomy, including the mandibular symphysis, appears to be a viable treatment option, however, short-term complications included clinically significant bleeding and species-specific features that increase the risk of surgical site trauma. We can conclude that en-bloc resection of a giant cell tumor of bone in a goat can be successfully performed, resulting in secondary fusion of both mandible with acceptable cosmetic and functional outcome.

## Data availability statement

The raw data supporting the conclusions of this article will be made available by the authors, without undue reservation.

## Ethics statement

Ethical review and approval was not required for the animal with the local legislation and institutional requirements. Written informed consent was obtained from the owner of the goat for publication of this case report and any accompanying images.

## Author contributions

NB: Conceptualization, Data curation, Investigation, Methodology, Writing – original draft, Writing – review & editing, Resources, Visualization. SP: Resources, Writing – original draft, Writing – review & editing. ND: Investigation, Visualization, Writing – original draft, Writing – review & editing, Data curation, Methodology. AF-B: Data curation, Methodology, Visualization, Writing – original draft, Writing – review & editing, Investigation. CW-L: Data curation, Methodology, Visualization, Writing – original draft, Writing – review & editing, Investigation. CB: Conceptualization, Data curation, Investigation, Methodology, Resources, Visualization, Writing – original draft, Writing – review & editing.
